# Supplementary material for: Effect of Aruncus dioicus var. kamtschaticus Extract on Neurodegeneration Improvement: Ameliorating Role in Cognitive Disorder Caused by High-Fat Diet Induced Obesity
Source: Nutrients. 2019 Jun 12;11(6):1319. doi: 10.3390/nu11061319 (PMC6628174; doi:10.3390/nu11061319)
Supplement: Supplementary file 1 [file nutrients-11-01319-s001.pdf]

Supplementary File

Table S1. Effect of ethyl acetate fraction from from *Aruncus dioicus* var. *kamtschaticus* (EFAD) on body weight and food intake of HFD-induced obese mice.

	**Control**	**HFD**	**EFAD20**	**EFAD40**
Initial body weight (g)	22.46 ± 1.21[a]	23.00 ± 1.10[a]	22.91 ± 1.81[a]	23.64 ± 2.45[a]
Final body weight (g)	31.14 ± 1.07[c]	49.38 ± 2.83[a]	45.71 ± 2.29[b]	45.43 ± 2.51[b]
Food intake (g/week)	26.18 ± 1.74[a]	18.35 ± 0.84[b]	18.18 ± 1.64[b]	17.73 ± 1.93[b]
Energy intake (kcal/week)	100.77 ± 6.71[a]	96.15 ± 4.40[a]	95.24 ± 8.60[a]	92.88 ± 10.11[a]

The data were statistically considered at $p < 0.05$ when comparing with the control group and statistical differences were represented as different small letters.
